# A refined TTC assay precisely detects cardiac injury and cellular viability in the infarcted mouse heart

**DOI:** 10.1038/s41598-024-76414-w

**Published:** 2024-10-24

**Authors:** Zheheng Ding, Xueqing Liu, Hongyan Jiang, Jianfeng Zhao, Sebastian Temme, Pascal Bouvain, Christina Alter, Puyan Rafii, Jürgen Scheller, Ulrich Flögel, Hongtao Zhu, Zhaoping Ding

**Affiliations:** 1 Institute of Biochemistry and Molecular Biology II, Medical Faculty and University Hospital Düsseldorf, Heinrich Heine University of Düsseldorf, Düsseldorf, Germany; 2 Department of Cardiology, The People’s Hospital of Danyang, Affiliated Danyang Hospital of Nantong University, West Xinmin Rd. 2, Danyang, 212300 China; 3 Institute of Anesthesiology, Medical Faculty and University Hospital Düsseldorf, Heinrich Heine University of Düsseldorf, Düsseldorf, Germany; 4 Institute of Molecular Cardiology, Medical Faculty and University Hospital Düsseldorf, Heinrich Heine University of Düsseldorf, Universitätsstr. 1, 40225 Düsseldorf, Germany

**Keywords:** Area-at-risk (AAR), Cardioprotection, CBB, redTTC, Myocardial infarction, Myocardial infarct size (mIS), Cardiovascular diseases, Cardiomyopathies, Mesenchymal stem cells

## Abstract

Histological analysis with 2,3,5-triphenyltetrazolium chloride (TTC) staining is the most frequently used tool to detect myocardial ischemia/reperfusion injury. However, its practicality is often challenged by poor image quality in gross histology, leading to an equivocal infarct-boundary delineation and potentially compromised measurement accuracy. Here, we introduce several crucial refinements in staining protocol and sample processing, which enable TTC images to be analyzed with light microscopy. The refined protocol involves a two-step TTC staining process (perfusion and immersion) and subsequent Zamboni fixation to differentiate myocardial viability and necrosis, and use of Coomassie brilliant blue to label area-at-risk. After the duo-staining steps were completed, the heart sample was embedded and sliced transversally by a cryostat into a series of thin sections (50 µm) for microscopic analysis. The refined TTC (redTTC) assay yielded remarkably high-quality images with striking color intensity and sharply defined boundaries, permitting unambiguous and reliable delineation of the infarct and area-at-risk. In the same animals, the redTTC assay showed good agreement with the in-vivo gold standard measurements (LGE and MEMRI). Meanwhile, redTTC imaging allows tracking of viable cardiomyocytes at cellular resolution, and with this enhanced capability, we convincingly demonstrated the pro-survival action of stem cells based-therapy. Therefore, the redTTC assay represents a significant technical advance that permits precise detection of the true extent of cardiac injury and cardiomyocyte viability. This approach is cost-effective and may be adapted for use in diverse applications, making it highly appealing to many laboratories performing ischemia/reperfusion injury experiments.

**Supplementary Information:**

The online version contains supplementary material available at 10.1038/s41598-024-76414-w.

## Introduction

Salvage of the infarct-jeopardized myocardium in the setting of acute myocardial infarction (MI) remains an unfulfilled promise, yet a highly desired therapeutic goal. The concept of cardioprotective therapy has been appreciated for more than 3 decades, during which numerous interventions, both pharmacological or physical, have been experimentally validated^1^. For studies aimed at mitigating ischemic injury, myocardial infarct size (mIS) has been considered a key scientific readout for assessing the success of novel therapies^2^. In this context, valid and reliable detection of mIS is critical and essentially required in translational research.

However, accurately assessing the true extent of myocardial injury is a daunting and challenging task. In animal models of MI, postmortem myocardial infarct size (mIS) is commonly determined by a histological assay based on 2,3,5-triphenyltetrazolium chloride (TTC) staining^3^. TTC is a colorless compound that is converted by active mitochondrial dehydrogenases to red precipitates in living tissue, while it remains as light gray in the area of necrosis^4^. The staining steps typically involve tissue sectioning into fine slices at a thickness of 1 mm and sequentially immersing the slices into neutral TTC solution for 15 ~ 20 min at 37 °C^2^. Once completed, the slices are scanned or photographed, and mIS is planimetrically calculated as a percentage of the left ventricular area^5^.

TTC staining, although broadly used as a gold-standard method, bears several intrinsic limitations, particularly in small-sized heart samples. Tissue slicing, while relatively easy in large animals, such as rabbit and swine, is rather difficult to execute in the murine^2^, leading to the transverse sections often uneven and nonuniform in thickness. To overcome this limitation, several refinements have been introduced to improve the practicality in tissue preparation. This includes the use of an acrylic heart matrix with nine parallel razor blades^6^ or embedding the heart in agarose^7^, and recently semi-freezing the heart to facilitate tissue slicing^8^. Nevertheless, these approaches are still strongly affected by the precise sequence and conditions in which the individual steps are carried out. As a result, the image quality varies widely and is often poor in terms of color contrast and boundary definition, which potentially introduces considerable subjectivity to mIS measurement and compromises the accuracy of analysis and even the validity of conclusions^8^. Thus, a reliable and standardized protocol to assess myocardial injury is critical for comparing the outcomes of putative cardioprotective interventions across laboratories and animal models and has become particularly desirable with the emergence of regenerative medicine, such as stem cells and cell cycle initiators^9^.

To tackle this challenge, we have scrutinized each step involved in the staining protocol and elaborated a refined approach that enables TTC images to be analyzed with light microscopy. The refined TTC assay (redTTC) consistently yielded high-quality images with stark color intensity and sharply defined boundaries, permitting unambiguous and reliable delineation of the infarct zone and the area-at-risk (AAR) in the infarcted mouse heart. Meanwhile, redTTC allows tracking viable cardiomyocytes at cellular resolution and can be adapted for use in diverse applications, from molecular signaling research to cellular transplantation studies.

## Materials and methods

This study was approved by the Institutional Animal Care and Use Committee at the Nantong University, China and by the Landesamt für Natur, Umwelt, und Verbraucherschutz Nordrhein-Westfalen, Germany. All animal experiments were conducted in accordance with ARRIVE guidelines and the NIH Guide for the Care and Use of Laboratory Animals. A total of 86 C57BL/6J mice (male, body weight 20–25 g, purchased from Janvier) were used in the present study, and all animals were fed a standard chow diet and tap water ad libitum.

### Experimental model of myocardial infarction

The mouse MI model was created by transiently occluding the left anterior descending (LAD) coronary artery, as previously described^10^. In brief, the animals were intubated and mechanically ventilated with gas mixture (30% O_2_ + 70% N_2_), containing 1.5% v/v isoflurane (Shandong Keyuan, China). The operation was performed on a pre-warmed operating table (37 °C) with a pair of electrodes linked to an electrocardiograph (ECG, lead II, ADInstrument). A thoracotomy was made in the fourth intercostal space, from the lateral side of the sternum to expose the LAD. A polypropylene suture (8–0 Prolene®, Ethicon) with a tapered needle was passed underneath the LAD, marginally below the tip of the left auricle and tied off firmly to stop the coronary blood flow downstream of the occlusion site. Myocardial ischemia was confirmed by both visual inspection of surface blanching and the immediate elevation of the ST segment in ECG registration. Fifty minutes later, the LAD occlusion was removed to ensure coronary reperfusion. The chest was then closed with one layer through the muscle and a second layer through the skin. The animals were weaned from ventilation and placed in a warm, oxygen-enriched environment until physical activity was fully recovered. Each animal received an intraperitoneal injection of butorphanol (1 mg/kg, Hengrui, China) for analgesic treatment.

### In-vivo detection of infarct size by MRI

In order to make a head-to-head comparison of the redTTC assay to the gold standard of in-vivo detection, we implemented noninvasive mIS measurement by using magnetic resonance imaging (MRI) performed longitudinally in the same animal^11^. In detail, the MI operation was scheduled in the morning, and the late gadolinium enhancement (LGE) experiment was sequentially carried out in the afternoon, about 6 h after the operation. The next morning before the mice being sacrificed, manganese‐enhanced MRI (MEMRI) was performed (about 22 h post-MI). For LGE experiments, the animals received an intraperitoneal injection of the contrast agent (Gd-DTPA, 0.1 mmol per kg BW), and for MEMRI, intravenous injection of a mixture of MnCl_2_ (60 µl, 0.01%) with calcium gluconate (25 µl, 0.03%) shortly before measurement. Cardiac images were acquired with the Bruker Microimaging unit (Mini 0.5) equipped with a 30 mm birdcage resonator. Six to eight contiguous ventricular short-axis slices (thickness = 1 mm) were acquired for each heart and all data were stored for offline analysis performed using the region of interest (ROI) tool in the Bruker ParaVision 5.1. The positive area in LGE (white) or negative area in MEMRI images (dark), as well as the left ventricle (LV) mass, were manually delineated at the end-systolic phase of cardiac cycles. The total infarct volume of each heart was calculated and multiplied by the slice thickness, and mIS was presented as a percentage of the infarct volume relative to the total LV mass. All the mice showed a quick recovery of physical activity shortly after surgery and MRI measurements, indicating that the mice well tolerated the operative stresses and subsequent measurements. There were no drop-offs of MI mice undergoing MRI measurement.

### TTC staining

Conventional TTC staining (conTTC) was performed according to the protocol previously reported^8^. In brief, the animal was euthanized by cervical dislocation under deep anesthesia (2.5% v/v isoflurane) shortly after MEMRI measurement. The heart was excised and washed once by retro-perfusion of cold PBS (Sigma-Aldrich, Germany) to remove residual coronary blood. The heart was placed on an iron plate with a drop of OCT compound (Tissue-Tek®, Germany) so that the tissue quickly adhered to the plate. After a brief cooling process at −80 °C (15 min), the semi-frozen heart was cut into 8–9 slices (~ 1 mm in thickness) by a razor blade. All tissue slices were placed separately in a 24-well plate filled with freshly prepared TTC solution (Sigma-Aldrich, Germany, 1% in PBS) and incubated at 37 °C for 20 min to allow color development. Once stained, the slices were fixed with 4% Paraformaldehyde (PFA, Sigma-Aldrich, Germany) and photographed using a digital camera (Nikon Z6).

To perform microscopic analysis in the refined TTC assay, we introduced several crucial modifications in the staining process and sample preparation based on a perfusion protocol^12^. These include two-step TTC color development (perfusion and immersion) and cryosectioning the tissue sample into thin slices.

**Fig. 1:**
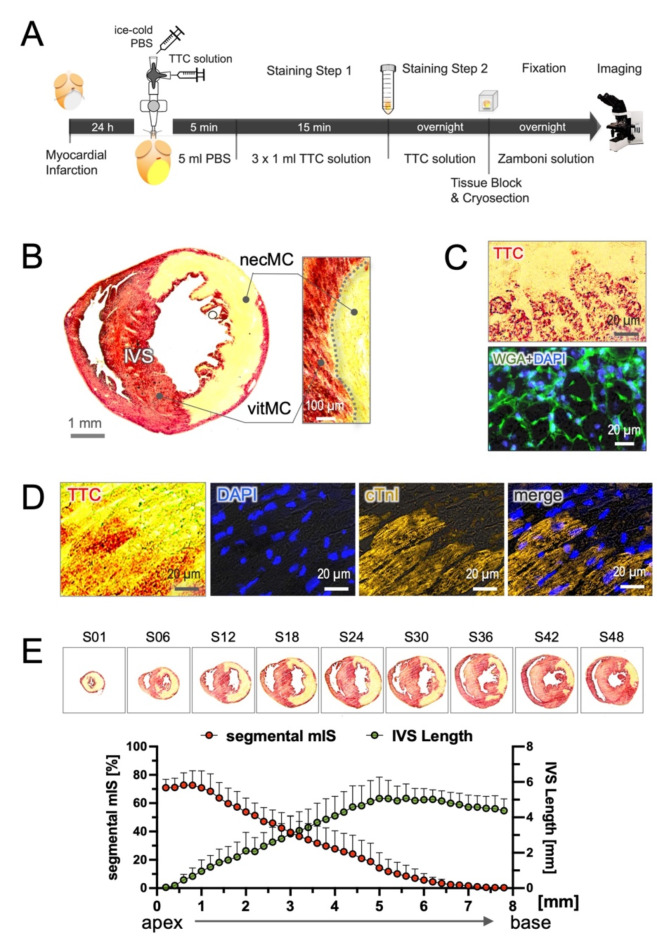
Microscopic TTC assay defines myocardial viability at cellular resolution. (**A**) Schematic of experimental protocol showing 2-step staining and cryosection for microscopic imaging. (**B**) A representative microscopic TTC image at mid-ventricular level of the 24-h MI heart, demonstrating two distinct areas with clear and unequivocal borderline: TTC-positive (deep red) indicates the vital myocardium (vitMC) and the picric acid-stained beige the necrotic myocardium (necMC). (**C**) At the cellular level, formazan precipitates were found in the cytoplasmic space of the viable cardiomyocytes figured out by WGA staining (green). (**D**) The formazan signal overlaps with viable cardiomyocyte, as defined by positive staining for cardiac troponin I (cTnI). (**E**) Quantitative analysis of segmental myocardial infarct size (mIS) in successive sections reveals dynamic variation in each slice, showing the greatest value in the apex and the lowest at the site of ligation (n = 5). The length of interventricular septum (IVS) correlated proportionally to the distance above the apex (n = 5), suggesting IVS may be considered as an internal landmark to reference the anatomical level of individual section.

In detail, the heart, along with the lung and surrounding tissue, was immediately excised from the thorax of mice 24 h after MI (or at extended time points of 3, 7, 28 days post-infarction, dpi). The entire tissue mass was washed once with ice-cold PBS and placed in a flat position on a Petri dish (150 × 15 mm). The aortic root was dissociated from the connective tissue and mounted onto a blunted 20G cannula in a retro-perfusion system (Fig. 1A), with the tip beneath the branch of the right carotid so that it was safely distal to the aortic valve. The cannula was secured in position with a single knot using 5–0 silk suture (SeraFlex®, Germany) and the heart was retro-perfused with 5 ml of ice-cold PBS at a rate of 1.5–2.0 ml/min to completely flush away the residual blood until the eluate from the coronary sinus drained clear. Subsequently, 1 ml of freshly prepared neutral TTC solution (1% in PBS) was infused into the coronaries and repeated three times every 5 min (3 × 1 ml). After the initial perfusion step (15 min), the heart was separated from the rest tissue (mainly including the lung and thymus) and transferred into a 15 ml Falcon tube containing 3 ml of TTC solution. The tube was placed on ice overnight for the second round of incubation. After the two steps were completed, both ventricular cavities were carefully filled with OCT compound via an atrial cannula and embedded in a vertical position in an OCT block with the help of a cooling bath filled with 2-methylbutane (-30 to—40 °C). The tissue block was stored at −80 °C for histological analysis.

### Microscopic imaging of TTC staining

Cryosections were made transversally at a thickness of 50 µm with intervals of 100 µm, covering the entire myocardium from the apex to the base of the heart. Approximately 80–85 sections were obtained from each heart and were sequentially arrayed on glass microscopy slides (8 samples per slide). The sections were desiccated using a hair dryer and fixed with Zamboni solution (Morphisto, Germany) at room temperature for at least 2 h or at 4 °C overnight (Fig. 1A). The fixation process efficiently removed faint background staining potentially caused by heme-containing proteins such as hemoglobin, cytochrome c, and myoglobin^13^, and picric acid in Zamboni solution additionally depicted the necrotic tissue in beige. After fixation, the slides were washed once with PBS and mounted with Aquamount (Polysciences, USA) and cover slips. All slides were photographed with a phase-contrast microscope (Olympus MX61, Japan) using a × 0.63 objective. Images were captured with a digital camera (UC30, Olympus, Japan) operated by CellSence® software (Olympus, Japan). From each image acquired, both myocardial infarct size (mIS) and myocardial viability were analyzed using ImageJ software (version 1.53a, NIH, USA).

### Calculation of myocardial infarct

#### Conventional mIS measurement

Myocardial infarct derived using conTTC method was based on a weight-based protocol. In brief, the stained tissue slices were manually dissected into two parts under stereomicroscopy: infarcted myocardium (TTC negative, grey) and viable myocardium (TTC positive, deep red). After excluding the right ventricular part, both portions of each slice were gathered and weighed. The percentage of the total infarct weight relative to the LV mass (both TTC negative and positive) was defined as myocardial infarct size (mIS).

#### Planimetric mIS measurement

An area-based approach was used to calculate mIS in each section^3^. First, the total area of the LV myocardium was tracked manually, and the infarct region (beige) was automatically contoured with the aid of the color-threshold tool and the ROI manager function in ImageJ software. The selected regions were primarily derived from pixel counts and converted to absolute values of area (µm^2^) after referring to the known length of the scale bar. Planimetric mIS, also known as segmental mIS, was then calculated by dividing the infarct area by the total LV area in the corresponding section.

#### Volumetric mIS measurement

**Fig. 2:**
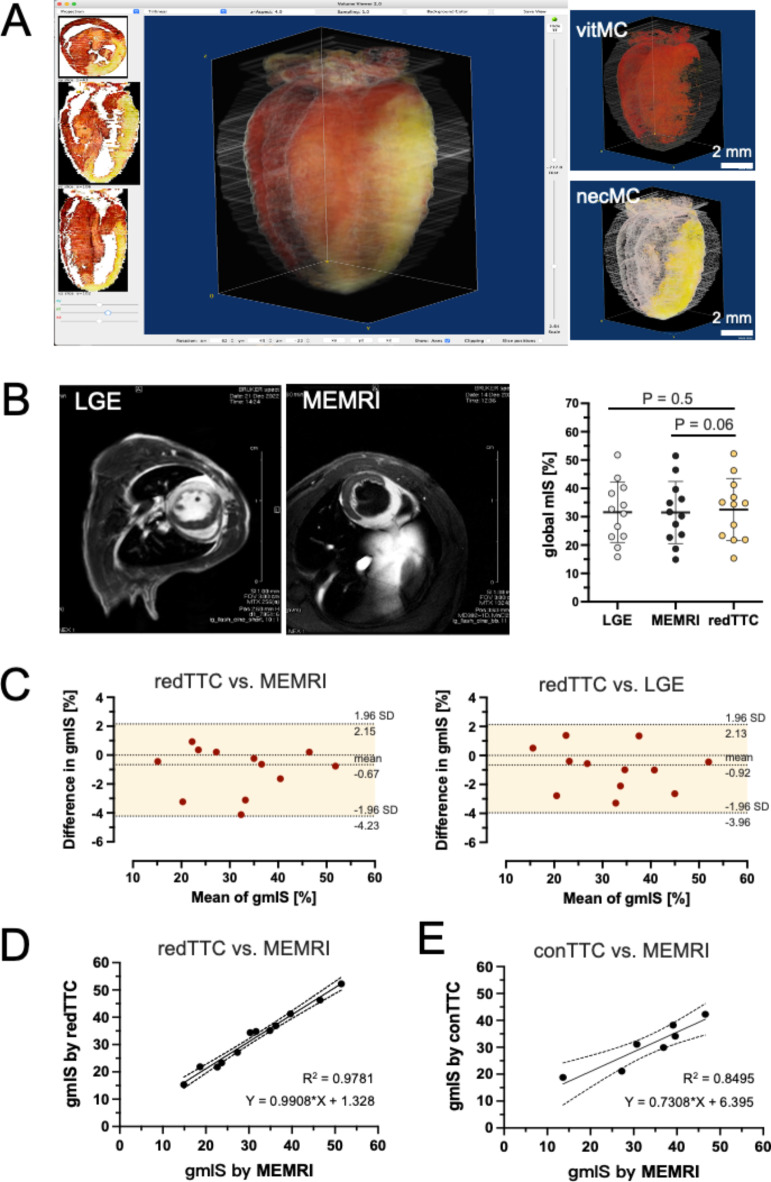
Validation and comparison of redTTC assay to in-vivo and conventional methods. (**A**) With computer-aided volumetry, 3D reconstruction was generated by interactive surface plotting using ImageJ software. The bulk infarct (beige) from the entire heart was calculated as the global mIS. **(B**) The global mIS was compared in the same animals to the infarct size derived by in-vivo methods: namely, late gadolinium enhancement (LGE) and manganese‐enhanced MRI (MEMRI) (n = 12). Within a broad range of infarct size (15 – 52%), no significant difference was detected (paired *t*-test). (**C**) Bland–Altman analysis shows good agreement in the measurements of redTTC to MEMRI (left panel) or LGE images (right panel) methods. (**D**) The mIS derived by the redTTC assay was regressed against the values of MEMRI images (y = 0.99x + 1.33, n = 12) and both groups demonstrates a significant correlation (R^2^ = 0.98, p < 0.0001, n = 12). The dashed line indicates 95% confidence interval (CI). (**E**) The mIS derived by conventional TTC method (conTTC) also showed a significant correlation to the values of MEMRI images (y = 0.73x + 6.40, R^2^ = 0.85, n = 7, p < 0.01). When comparing both correlation coefficients, the redTTC assay shows significantly closer relation to MEMRI measurements, as compared to the conTTC method (*z*-test, p < 0.001).

A volume-based approach was used to calculate mIS on the basis of the entire heart. To create a 3D projection, images of individual sections were carefully aligned, and 3D reconstruction was generated by interactive surface plotting using the add-in tool in ImageJ software. Volumetric mIS was calculated by dividing the sum of the pixels of infarcted area (beige) by the total pixels of the LV volume, which included both viable myocardium (vitMC) and necrotic myocardium (necMC) in the 3D projection (Fig. 2A). Volumetric mIS, or global mIS, was expressed as the percentage of the infarct volume relative to the holistic LV mass.

#### IVS Length measurement

Length of the interventricular septum (IVS) represents the segmentary span of the curved myocardium that partitions the left and right ventricles. It correlates proportionally to the distance from the apex and can be used as an internal reference to define the anatomical level of sections. To calculate the IVS length, both margins at the anterior and posterior interventricular sulci were manually defined and the segmentary line of the IVS mid-wall was computed using ImageJ software. The length was normalized to the scale bar and expressed as absolute value in millimeter.

### Determination of area-at-risk

Area-at-risk (AAR) stands for the part of myocardium jeopardized by ischemia and is usually calculated as the area distal to the tissue with potent perfusion, known as remote myocardium (RM). In animal experiments, RM is frequently determined by infusion of water-soluble dye, typically Evan’s Blue^14^ or Phthalocyanine blue^8^ to depict the myocardium with unobstructed blood flow. In the present study, we employed Coomassie Brilliant blue R250 (CBB, Carl Roth, Germany) as a feasible candidate that stably labeled the RM without smearing particularly in cryo-processing the cardiac tissue. In brief, CBB powder was prepared at working concentration of 1% (w/v) in saline and, to remove undissolved particles, the solution was once spined down at 20000 rpm for 20 min and filtered with a syringe Filter (0.45 µm, Minisart®, Sartorius, Germany) shortly before use. CBB solution (300 µl) was administrated into the coronaries via the aortic cannula, shortly after the initial step of TTC staining (perfusion), when the LAD was re-occluded using the preserved suture in situ to anatomically match the initial occlusion in vivo. The heart was washed once with PBS and placed in TTC solution overnight for the second round of staining as mentioned above. After the two rounds of staining were completed, the heart was embedded in OCT compound for cryosection. Microscopic imaging was performed in the same way as described above.

To compare CBB approach with the conventional method using Evan’s blue, the MI heart was harvested and rinsed with 5 ml of ice-cold PBS via the aortic cannula. The LAD was then re-occluded, and 300 µl of Evan’s blue solution (2%, Morphisto, Germany) was given intracoronarily as previously reported^3^. After washing once with PBS, the heart was proceeded according to the standard protocol of conventional TTC staining and gross histology, as mentioned above (Suppl. Figure 3A).

**Fig. 3:**
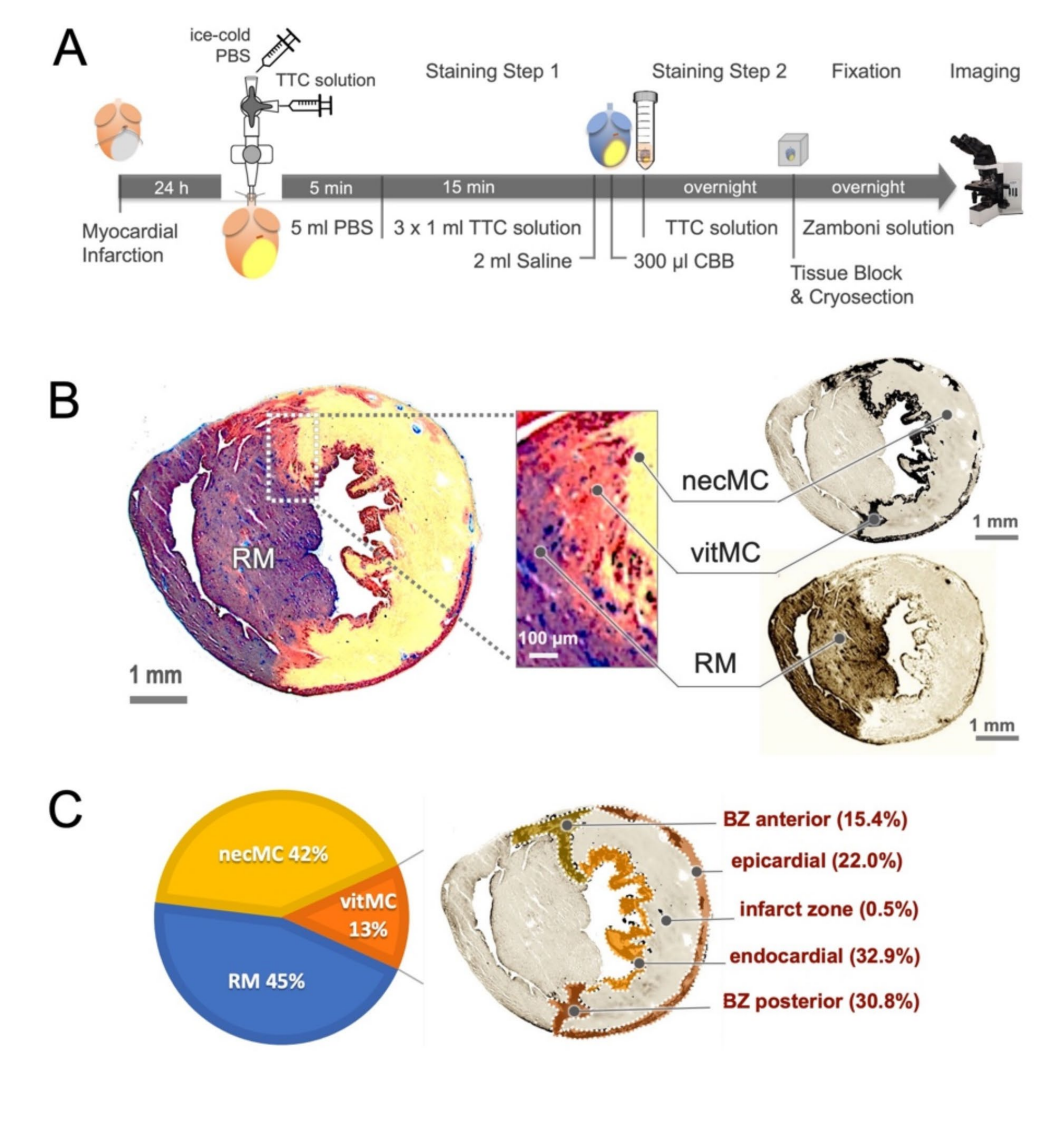
Measurement of mIS:AAR ratio. (**A**) Schematic drawing of staining protocol for TTC and CBB double-staining. (**B**) Microscopic imaging of the double-staining section reveals that CBB markedly stains the remote myocardium (RM) blue. AAR is computed as the area distal of the RM, and the vital myocardium (vitMC in red) and the necrosis (necMC in beige) are separated into RGB single channels using a color-threshold tool. (**C**) The portions of RM, necMC and vitMC are calculated in the mid-ventricular section from 5 hearts (n = 5). Within the AAR, vitMC is found to predominantly locate in the epicardial, endocardial, anterior border zone (BZ) and posterior BZ regions, with the least found in the infarct zone (0.5%).

To calculate AAR, the LV area was manually contoured in the double-stained sections, and three tinctorial areas were identified and split into RGB single channel using a color-threshold tool (blue = RM, beige = mIS, and red = vitMC). The viable cardiomyocytes in the AAR were further assigned into 5 distinct regions upon the locations: epicardial, endocardial, border zone (BZ) anterior, BZ posterior, and infarct zone (Fig. 3C). The pixel counts as well as the absolute value of area (mm^2^) in each channel were automatically planimetered with the aid of ROI manager function in ImageJ software. The segmental mIS:AAR ratio was presented as the infarct area dividing the value of AAR in the corresponding section. Global mIS:AAR ratio was computed by dividing the bulk infarct (sum of each slice) by the total AAR volume. Both segmental and global mIS:AAR ratios were expressed as a percentage multiplying by 100.

### Immunostaining in heart sections

Immunostaining was performed on the heart samples that had undergone two-step TTC staining. In brief, cryosections were made in 10-µm thickness from the tissue block and fixed with 4% paraformaldehyde (Sigma Aldrich, Germany) for 10 min at room temperature. The following primary antibodies were used in the present experiments: mouse anti-α-actinin (Thermo Fisher, USA), rabbit anti-CD31 (Proteintec, USA), rabbit anti-cardiac troponin I (Proteintec, USA), rat anti-laminin (abcam, USA) and rabbit anti- caspase-3 (Casp-3, abcam, USA), rat anti-CD45 (Ab5, Neomarker, USA) and rabbit anti-Periostin (POSTN, OriGene, USA). All antibodies were diluted at a ratio of 1:200 and incubated with the tissue sample at 4 °C overnight. Next day, the slices were washed 3 times with PBS and further incubated with the secondary antibodies (Cy3- or FITC-conjugated goat anti-rabbit or rat, 1:400 and Cy3-conjugated goat anti-mouse Fab IgG, 1:400) or wheat germ agglutinin (WGA, 1%, Thermo Fisher, USA) at room temperature for additional 2 h. For α-actinin staining, blockage with Fab IgG saturation or normal goat serum was used to minimize cross reaction. Cell nuclei were counterstained with 4’,6-diamidino-2-phenylindole (DAPI), and slides were mounted with Prolong™ Gold (Thermo Fisher, USA). Images were acquired using a fluorescence microscopy (Olympus MX61, Japan).

### Determination of myocardial survival in the infarct zone

Given the accuracy and high sensitivity of the redTTC assay in detecting cellular viability, we revisited the cardioprotective effects of stem cell-based therapy. In this context, we isolated pericardial adipose stem cells (ADSC) from a transgenic lineage as previously reported^15^, in which reporter gene (eGFP) was driven by the expression of Wilms’ tumor factor 1 (WT1CreERT/eGFP^+^). ADSC were enzymatically isolated from the pericardial tissue of 5-day post-MI mice that had received two injections of tamoxifen (1 mg/day i.p.). The cells were cultivated in low sugar Dulbecco’s modified Eagle’s medium (DMEM, Sigma) supplemented with 30% FCS (PAN Biotech), penicillin (100 U/ml, Sigma), streptomycin (0.1 mg/ml, Sigma), and glutamine (1 mM, Sigma) and underwent fluorescence-activated cell sorting (FACS, Canto™II, BD) to obtain a pure progeny of the WT1-expressing cells (ADSC^WT1/eGFP+^). The eGFP negative population (ADSC^WT1/eGFP-^) was used as control.

For isograft cell transplantation, intramyocardial injection was made immediately after the onset of reperfusion by using a 30 G U-100 insulin syringe of (Micro-Fine, BD). Cells were resuspended in 40 µl PBS and injected into the infarcted ventricular wall at 4 sites with a total number of 100,000 cells^16^. After the injection, the chest was closed with one suture through the muscle and a second through the skin, and the animals were allowed to fully recover until hemodynamics stabilized.

Detection of viable cardiomyocytes using the redTTC assay was performed 24 h after injection as described above. The surviving cardiomyocytes, indicated by TTC-positive signals within the infarct area, were contoured automatically using ImageJ software and summarized from the slice number 5 to number 30, where 4-site myocardial injections were primarily targeted. The survival rate of cardiomyocytes was presented as the percentage of bulk viable cardiomyocytes (TTC-positive) relative to the total infarct area in the corresponding sections.

### Statistical analysis

Data were presented as mean ± standard deviation. All experimental data were subject to D’Agostino’s K-squared test to assess normality. A Student *t*-test with Welch’s correction was used to compare the survival rate of cardiomyocytes following the injections of the two types of cells. Comparison of mIS derived by LGE, MEMRI, and the redTTC assay in the same animals was performed using a paired *t*-test. Agreement of the mIS values derived from the redTTC assay and in-vivo measurements using LGE and MEMRI techniques was tested using Bland–Altman analysis. The correlation coefficients between redTTC vs. MEMRI and conTTC vs. MEMRI were analyzed using a *z*-score test. Differences were considered significant at p < 0.05. Statistical analyses were conducted using the Prism software package (version 9.0).

## Results

### Detection of myocardial injury

The most crucial refinement in the present protocol involves infusing neutral TTC solution into the heart and to proceed the tissue sample into thin cryosections, allowing for imaging with a light microscopy (Fig. 1A). This approach, previously employed in our lab to analyze neutrophil infiltration in the injured heart^17^, is now systemically evaluated for its applicability in analyzing myocardial injury in mouse hearts. At microscopic resolution, formazan precipitates were primarily observed in the cytoplasmic space of viable cardiomyocytes figured out by membrane staining with wheat germ agglutinin (WGA, Fig. 1C). The specific cardiomyocyte labeling was also confirmed by immunostaining with cardiac troponin I (cTnI), which was rapidly degraded in the damaged cardiomyocytes within first 24 h after MI but persisted exclusively in viable cardiomyocytes (Fig. 1D). The intense deep-red coloration in the vital myocardium (vitMC) clearly distinguishes it from the beige staining of necrotic myocardium (necMC), creating a sharply defined border that precisely delineates the infarct zone (Fig. 1B) compared to the conventional TTC images (Suppl. Figure 1B). Furthermore, the redTTC assay differentiates the vitMC from living non-myocytes, such as immune cells and fibroblasts, which exhibit significantly weaker TTC signals in the heart sections (Suppl. Figure 2B). This distinctive feature suggests that the current method can label cardiomyocytes in an extended time frame beyond the acute phage (3–28 days after MI, Suppl. Figure 2A), and may serve as a valuable tool for analyzing cardiac myogenesis in chronic/old MI models.

Metameric sectioning of the entire heart yielded approximately 80 tissue slices at intervals of 100 µm. In most cases, slice number 1 (S01) to number 60 (S60) corresponded to the infarcted zone, while the remaining slices covered the tissue above the occlusion site. The values of mIS derived using the area-based method in each section, known as segmental mIS, was relatively variable. The highest values were observed at the apex (70–80%) and gradually decreased to zero near the site of ligation (Fig. 1E). This dynamic change suggests that when single mIS value is employed to evaluate the effectiveness of interventions aimed at limiting cardiac injury, comparison should be made on similar anatomical levels across different hearts.

In this context, we propose using the length of IVS as a useful anatomical reference. The IVS length correlates proportionally with the distance from the apex of the heart, starting from a minimum at the apex and reaching its maximum at the mid-ventricular level (Fig. 1E). This relationship suggests that IVS length can serve as an internal landmark to help identify the anatomical level of heart sections analyzed. This is particularly relevant in chronic/old MI models, in which precise anatomical location of individual sections can become difficult to determine due to significant cardiac remodeling and dilated cardiomyopathies. The use of IVS length may provide a consistent way to align sections with comparable anatomical levels and to more accurately compare results across heart samples or different studies.

The true extent of infarct was unbiasedly represented by the global mIS derived by volume-based method. With computer-aided volumetry, we calculated global mIS by the 3D-projection model reconstructed from all slides covering the holistic LV (Fig. 2A). The value of global mIS (32.53 ± 10.9%) was roughly comparable to the value of segmental mIS slightly below the mid-ventricular level (Suppl. Figure 4).

The global mIS measured by the redTTC was compared to the values derived by well-recognized in-vivo methods, namely LGE and MEMRI (Fig. 2B). Myocardial infarct was experimentally produced in a broad range of infarct size (15.32–52.22%), and mIS was assessed by three approaches in the same animal. The mIS values derived by individual method were similar and statistically insignificant (p > 0.05 by paired *t*-test, right panel in Fig. 2B). Bland–Altman analysis further demonstrated a good agreement between measurements by the redTTC and the in-vivo methods of MEMRI and LGE (limits of agreement =  − 0.67 ± 1.38% and − 0.92 ± 1.55%, respectively, Fig. 2C). The redTTC-derived mIS showed a perfect correlation with the mIS by MEMRI (y = 0.99x + 1.33, R^2^ = 0.98, p < 0.0001, Fig. 2D), while the conTTC method a compromised correlation to MEMRI assessment (y = 0.73x + 6.40, R^2^ = 0.85, p < 0.01, Fig. 2E). A *z*-test comparison of the correlation coefficients of two methods (redTTC and conTTC) with MEMRI revealed a significant difference (p < 0.01), suggesting the redTTC assay is more closely aligned with the in-vivo gold standard and exhibits less variation than the conventional method.

The most precise and reliable measure to depict the severity of cardiac injury frequently relies on the ratio of mIS:AAR, in which infarct is referred to the myocardium at risk of ischemia (AAR). To accurately determine AAR, CBB with the protein-binding property was used to effectively solve the problem of water-soluble dyes such as Evans blue, which is prone to smearing towards unstained myocardium particularly in the process of cryosectioning (Suppl. Figure 3B, left). As a result, CBB staining clearly outlined the RM with a stable and discernible borderline (Fig. 3B). At the microscopic resolution, CBB-stained area was found to cover mainly the septum and the right ventricle and as expected, about 13% of cardiomyocytes survived after ischemic episode, located in the anterior and posterior marginal regions at the peri-infarct border zone as well as in the endocardial and epicardial regions (Fig. 3C). We found that AAR also varied significantly depending on anatomical level of the sections and was by 7.2 mm^2^ at the apex and nearly doubled at the mid-ventricular level (Suppl. Figure 4). With a parallel increase in the infarct area, the mIS:AAR ratio remained relatively constant up to the mid-ventricular level and gradually declined until the site of occlusion (indicated by the bar in Suppl. Figure 4), mainly due to the collateral survival of cardiomyocytes (Suppl. Figure 4). The final and unbiased extent of infarct was presented as global ratio of mIS:AAR calculated by volumetry (the box and whiskers symbol in Suppl. Figure 4), showing that the value was slightly below the segmental ratio at the mid-ventricular level.

### Detection of myocardial viability

Cardiac benefit of stem cell-based therapy has recently been proposed to be mediated by paracrine factors that protect cardiomyocytes against ischemic stress^18^, yet this concept has not been convincingly proven. Detecting cardiomyocyte viability by gross histology is technically difficult at cellular resolution. Given the technical merits of the redTTC assay, we revisited the impact of cardiomyocyte survival on the cardiac benefits induced by stem cell-based therapy.

**Fig. 4:**
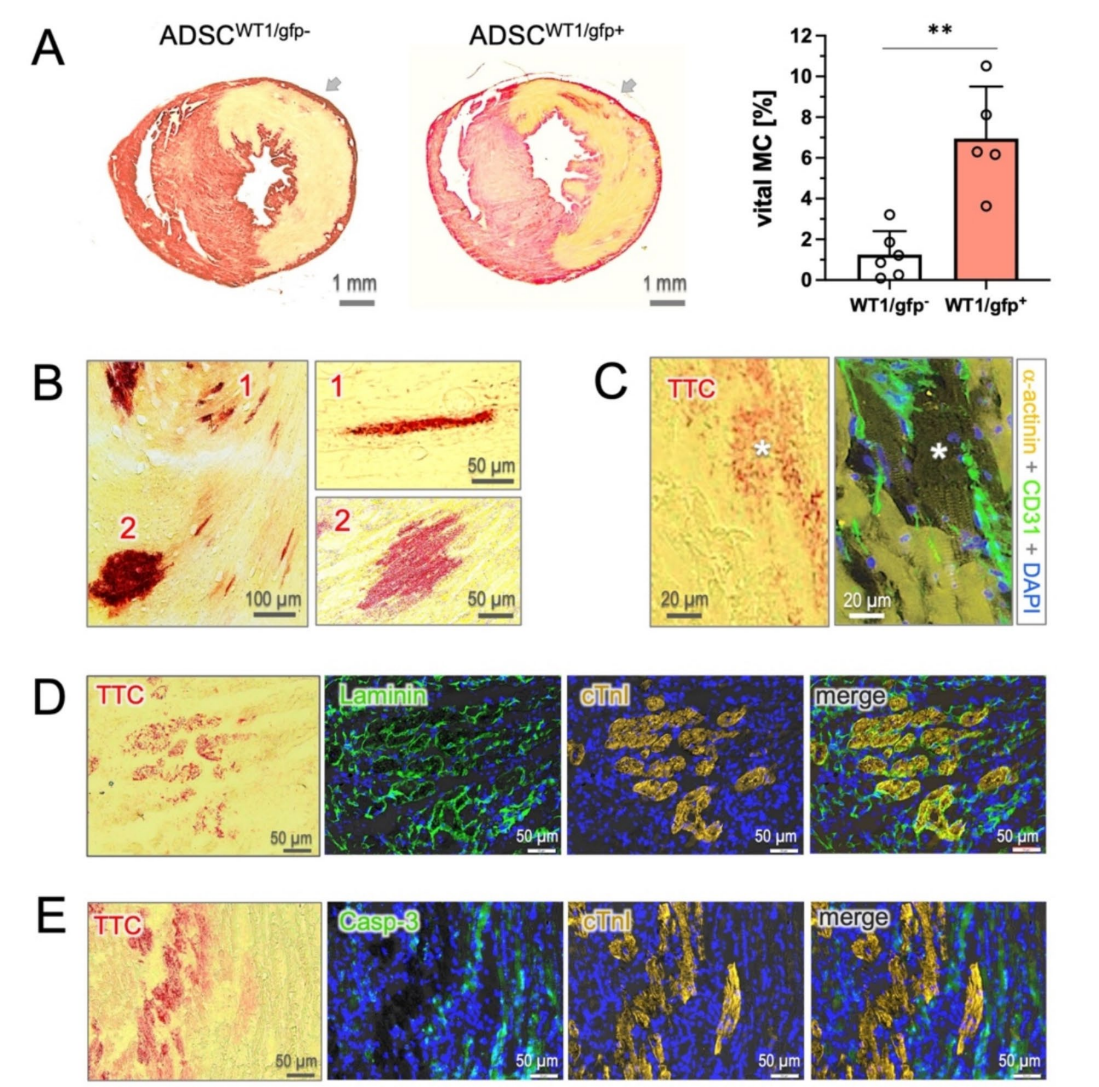
Detection of cardiomyocyte survival using redTTC assay. (**A**) Subtle islands of surviving cardiomyocytes are detected within the infarct zone. The vital myocardium (vitMC) is significantly more abundant in the hearts that received injections of active form of stem cells (ADSC^WT1/gfp+^, n = 5) compared to those injected with the dormant cells (ADSC^WT1/gfp-^, n = 6). Arrows indicate the injection site. (**B**) The vitMC survive either as single cells or in clusters within the infarct area. (**C**) TTC signal overlays precisely with cardiomyocytes, as identified by the typical striations in α-actinin staining (asterisk). Note that the necrotic cells also exhibit a high affinity to anti-α-actinin antibody. (**D**) TTC-positive cells retain cellular integrity, as shown by the intact membrane basement stained by laminin (green). (**E**) Positive casp-3 staining is primarily found in TTC-weak/negative cells, but not in TTC-dense cardiomyocytes, indicating an absence of apoptotic events in the surviving cardiomyocytes. ** indicates p < 0.01.

In the MI heart, vitMC was found to localize primarily in the endocardial and epicardial regions and peri-infarct zone, with a minor amount within the infarct area at the mid-ventricular level, (< 0.5%, Fig. 3C and Suppl. Figure 4). However, measurable subtle islands of vitMC were readily detectable within the infarct area where stem cells were usually injected (arrow in Fig. 4A). The pro-survival effect was significantly enhanced by injection of active form of ADSC (ADSC^WT1/gfp+^) compared to the dormant stem cells (ADSC^WT1/gfp-^, right panel in Fig. 4A, p < 0.01)^16^. At microscopic resolution, the viable cardiomyocytes were found to survive in clusters or as single cells with elongated structure typically seen in mature cardiomyocytes, excluding the possibility of artificial noise caused by cardiac hemorrhage (Fig. 4B). Co-localization of TTC-positive signal was confirmed by immunostaining, showing exact overlay with the viable cardiomyocytes characterized by typical striations of α-actinin staining (asterisk in Fig. 4C, also note the nonspecific binding in necrotic cells). The TTC-labeled cardiomyocytes exhibited the cellular integrity, demonstrated by the intact membrane basement in laminin staining (Fig. 4D) and, notably, negative for Casp-3 (Fig. 4E), suggesting the apoptotic absence in the surviving cardiomyocytes.

## Discussion

In the present study, we introduce a comprehensive, microscopy-based imaging strategy for TTC assay to elucidate cardiac lesion and cellular viability in infarcted hearts. This approach remarkably yielded high-quality images with stark color intensity and sharply defined boundaries, permitting unambiguous and reliable delineation of the infarct zone and AAR in the mouse heart. With these distinctive merits, we provided, for the first time, experimental evidence convincingly demonstrating the pro-survival action of stem cell-based therapy.

### Detection of myocardial injury by redTTC assay

TTC assay, also known as TTC test or tetrazolium test, is broadly used to differentiate between metabolically active and inactive tissues^19^. The white compound is enzymatically reduced to red formazan in living tissue upon the enzymatic activity of dehydrogenase and co-factors (NAD, NADP), while it remains as colorless in areas of necrosis where the enzyme has been either denatured or degraded^4^. This method has been considered as the gold standard to assess postmortem mIS in multiple animal models. In mice, staining steps typically include tissue section into fine slices (generally 1 mm thick) with the help of an acrylic heart matrix with nine parallel razor blades^6^, or by embedding the heart in agarose^7^ or briefly semi-freezing the sample to facilitate slicing^8^, and sequential immersion of the slices into TTC solution to tinctorially differentiate viable and nonviable myocardium. The major limitation of conventional TTC staining method, however, is that the image quality derived from the thick tissue slices is highly variable across laboratories and often poor in terms of color contrast and border definition, leading to considerable inaccuracy and, potentially, subjectivity in mIS measurement^2^.

In the present study, we meticulously scrutinized each step of the staining protocol and made several crucial refinements to improve image quality up to cellular resolution. The optimized approach involves a two-step staining process: transcoronary retro-perfusion of neutral TTC solution into the heart and subsequent immersion in TTC solution. The first infusion step warrants even and thorough distribution of TTC over the entire heart, and the second round of immersion is necessary to obtain the accurate and complete picture of the cardiac injury. This ensures proper color development and further tinction of the areas that have not been initially well perfused, particularly in the endocardial and epicardial regions (Suppl. Figure 1A). During the staining steps, the heart samples were kept at a low temperature (4 °C) to help maintain cell viability, which may minimize potential extension of injury caused by the staining process. In contrast, the warm conditions and the freezing/staining process used in the previously reported conTTC method^8^ may potentially cause tissue damage and affect the accurate mIS assessment. Taken together, the redTTC assay represents a significant technical advance, enabling the precise detection of the true extent of cardiac injury.

It is also important to note that tetrazolium when dissolved in saline (0.9% NaCl) has very low pH value (3.7), and the acidic solution inevitably causes severe cardiac contraction, preventing subsequent detection of AAR with CBB^8^. To avoid this side effect, a neutral TTC solution prepared in PBS is critically important. After two steps were completed, we proceeded the heart tissue into thin slices (10–50 µm) for the first time by a cryostat for microscopic analysis. This strategy improved substantially the quality of histological images compared to conventional methods (Suppl. Figure 1B). With the clear-cut and unambiguous boundaries, we evaluated segmental and global mIS. The value of global mIS was almost comparable to the mIS values derived by in-vivo methods (LGE and MEMRI) performed longitudinally in the same animal (Fig. 2B), and Bland–Altman analysis demonstrated good agreement with the values from gold standard methods (Fig. 2C). Thus, given its unbiased and highly reproducible detection of the true extent of myocardial injury, the redTTC assay may potentially be adapted as a streamlined tool in research on cardiac ischemia/reperfusion injury to minimize experimental noise and sample size.

It is important to note that, in an chronic/old MI heart beyond the acute phase, thinning of the infarcted segment and cardiac remodeling/dilated cardiomyopathies may have occurred^20^, leading to a substantial geometric alteration of heart structure and considerable enlargement of heart size. Determining infarct size in single-slice comparisons can be challenging^2^, as achieving a comparable level of section from different hearts is often difficult, even when the distance from the starting level of the apex is well controlled. These potential pitfalls in comparative analysis based on single-slice cast some doubt on the validity of mIS as a reliable readout and imply that the results should be cautiously interpreted. In this context, we propose IVS length as a potential internal landmark to reference sections at similar levels, which may provide a simple solution to minimize experimental noise in mIS comparisons.

The final extent of infarct size is expressed ideally as the ratio of mIS:AAR and, to this end, precise measurement of AAR is equally critical to the accuracy of infarct detection^3^. In previous publications, a water-soluble dye such as Evan’s blue, or phthalocyanine blue, was frequently used in various animal models. Evan’s blue is an azo dye that, when injected into the circulation, binds tightly to plasma albumin, and evenly distributes to the tissue with potent perfusion, staining that tissue dark blue. However, such pigment is prone to smearing out into unstained tissue, which often obscure borderline definition. In pilot experiments, we tested several candidates and identified CBB as a feasible pigment that stably labeled the RM blue with clear and unambiguous boundaries (Fig. 3B).

CBB is a water-soluble dye used to visualize protein bands in polyacrylamide gel primarily in biochemical studies^21^. It forms insoluble precipitates after interacting with proteins and thus stably labeling the tissues where it accesses. It is important to note that, during practical handling, CBB tends to form precipitates quickly in phosphate buffer and, therefore, CBB powder should be dissolved in saline, in which CBB remains water-soluble. Therefore, it is also necessary to cautiously flush TTC solution (in PBS) out of the perfusion system with saline before applying CBB to avoid in-vessel formation of precipitates. With this adaptation, the current CBB staining protocol is an easy-to-handle method that persistently labels the RM even after cryo-processing.

### Myocardial survival detected using the redTTC assay

Cardiomyocyte survival was found within regions deprived of blood flow (AAR), likely due to collateral blood supply in the marginal zone and oxygen diffusion to the endocardial region. The surviving myocardium accounts for about 13% of the total LV mass (Fig. 3C), which is moderate compared to 18% in a previous study using the conventional TTC method that showed a larger proportion of surviving cardiomyocytes in the posterior marginal zone^8^. Nonetheless, by both imaging techniques (conTTC and redTTC), cardiomyocyte survival within the infarct were often found to be rare, with a limited extent, representing only 0.5% of the LV mass (Fig. 3C).

Recently, the prevailing concept of stem cell efficacy has shifted toward the paracrine hypothesis, which proposes that transplanted cells produce soluble factors beneficial to the infarcted heart^16^, likely through a powerful effect of cellular postconditioning^18^. However, this hypothesis lacks experimental evidence convincingly demonstrating cardiomyocyte survival in the acute phase of infarction after cell transplantation. In the present study, our redTTC assay shows its merits in detecting viable cardiomyocytes at cellular resolution and provides compelling evidence that a sizable number of cardiomyocytes appreciably retain viability (TTC positive) within the infarct zone, particularly in hearts treated with an active form of stem cells (Fig. 4A)^22^. Cardiomyocytes were found to survive in clusters or as single cells that show structural integrity and an absence of apoptosis, suggesting that cardiomyocyte survival has indeed occurred in the setting of stem cell transplantation. Furthermore, with the unique feature of the redTTC imaging to consistently detect cellular viability, it may serve as a helpful tool to track viable cardiomyocytes and decipher the survival mechanisms in the fraction of cardiomyocytes that are highly resistant to ischemic stress.

### Strengths and weaknesses of the redTTC versus conTTC methods

The redTTC assay, in addition to generating remarkably high-quality images, identified viable cardiomyocytes at cellular resolution. Cardiomyocyte viability is usually difficult to be specified at the microscopic level with traditional histological methods. TTC labeling persisted in the tissue sections after several rounds of washing steps (Fig. 1D) and was preservable for more than one year when stored at 4 °C in our lab. Notably, TTC labeling persisted in the tissue sections after several rounds of washing (Fig. 1D) and remained stable for more than one year when stored at 4 °C in our lab. Moreover, the tissue retained epitope reactivity after TTC staining, allowing for successful combination with immunostaining with negligible background autofluorescence (Fig. 1D, Fig. 4D, E and Suppl. Figure 2B). This suggests the tissue samples can be utilized for multi-purpose studies.

However, we acknowledge that the redTTC assay has several weaknesses that may limit its widespread adoption. The primary concern is the time-consuming nature of the method. It seems that the entire procedure is relatively lengthy, requiring about three days for one round of preparation (Fig. 3A), compared to only about 1–2 h for the conTTC assay (Suppl. Figure 3A), Nevertheless, the majority of the time needed is spent on overnight incubation and fixation. In fact, sample-handling time required for both methods is by and large identical when the cryosection is done at intervals of > 500 µm and fewer intermediate slices are collected and analyzed (14–16 slices per heart). This may substantially reduce the size of multilayer image dataset and shorten the hands-on time needed without significantly impacting the accuracy of mIS measurement. Additionally, the estimated time can also be significantly averaged down when multiple animals are processed in parallel on the same day.

Additionally, the redTTC assay seems more suited for small-sized hears, such as those of rodents rather than large animals. However, given the increasing interest in using mice as experimental models for MI studies due to its genetic malleability and cost-effective husbandry, the redTTC assay presents itself as a highly promising method for many laboratories.

## Conclusions

In summary, the microscopic imaging of the TTC assay introduced in this paper represents several important technical advances that ensure unequivocal and accurate detection of myocardial injury and cellular viability. The redTTC assay is simple, cost-effective, and adaptable for use in diverse applications, from molecular signaling research to cellular transplantation studies. Therefore, we propose this method as a potentially impactful tool to precisely measure the true extent of ischemic injury and cardiomyocyte survival, which should be of great interest to many researchers performing myocardial ischemia/reperfusion injury experiments.

## Electronic supplementary material

Below is the link to the electronic supplementary material.

Supplementary Material 1

## Data Availability

All data supporting the findings of this study are available within the paper and its Supplementary Information.

## Abbreviations

AAR: Area-at-risk
ADSC: Adipose stem cells derived from pericardial tissue
CBB: Coomassie brilliant blue R250
necMC: Necrotic myocardium
vitMC: Vital myocardium
surCM: Surviving cardiomyocytes
mIS: Myocardial infarct size
IVS: Interventricular septum
LAD: Left anterior descending artery
LGE: Late-gadolinium enhancement
LV: Left ventricle
MEMRI: Manganese-enhanced magnetic resonance imaging
MI: Myocardial infarction
redTTC: Refined TTC assay
RM: Remote myocardium
ROI: Region of interest
TTC: 2,3,5-Triphenyltetrazolium chloride
WT1: Wilm’s tumor factor 1
