# Supplementary material for: A refined TTC assay precisely detects cardiac injury and cellular viability in the infarcted mouse heart
Source: Sci Rep. 2024 Oct 24;14:25214. doi: 10.1038/s41598-024-76414-w (PMC11502796; doi:10.1038/s41598-024-76414-w)
Supplement: Supplementary file 1 — Supplementary Material 1 [file 41598_2024_76414_MOESM1_ESM.docx]

**Supplementary Figures and Legends**

**
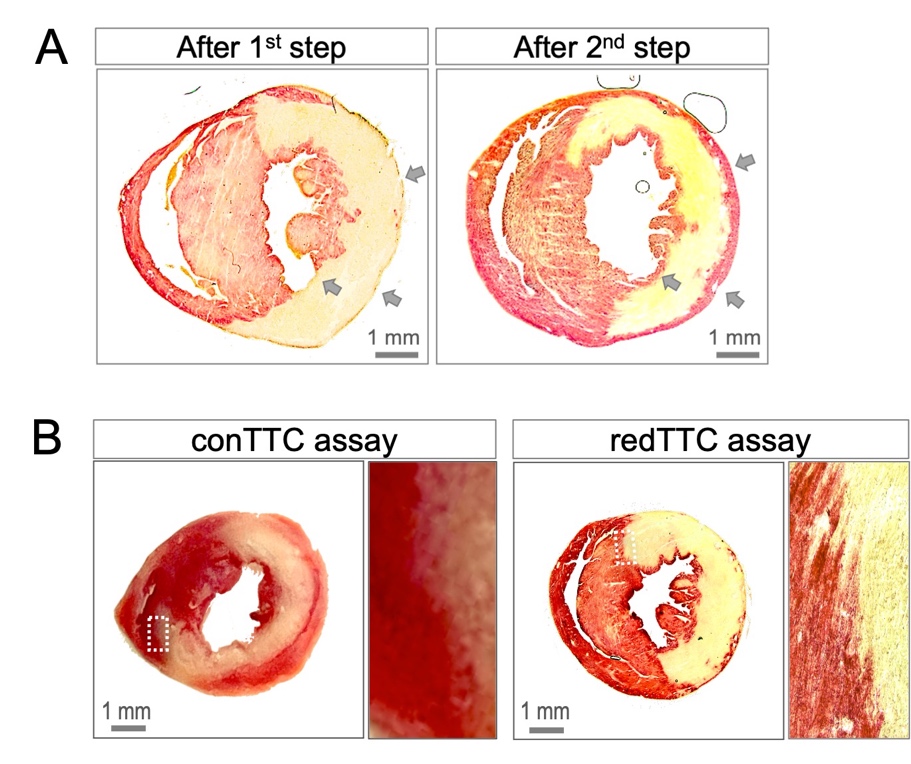
**

**Suppl. Fig. 1.** Validation of the two-step procedure in red TTC staining and comparison of the image quality between the two methods. **A.** In the refined TTC (redTTC) staining protocol, the heart was stained in perfusion step in the first round (left) and followed by the second step of immersion (right). After the two-step staining was completed, the heart section showed further tinction in the endocardial and pericardial regions (arrows), which was not observed in the heart sections only after the first round. **B.** A head-to-head comparison was conducted using either conventional TTC staining (conTTC) with gross histology at a thickness of 1 mm, or by the redTTC method with thin section (50µm). The conTTC images showed an uneven section with blurry borderline of the infarct (insert), and the redTTC assay was characterized by a clear-cut, unambiguous boundary at cellular resolution (insert).


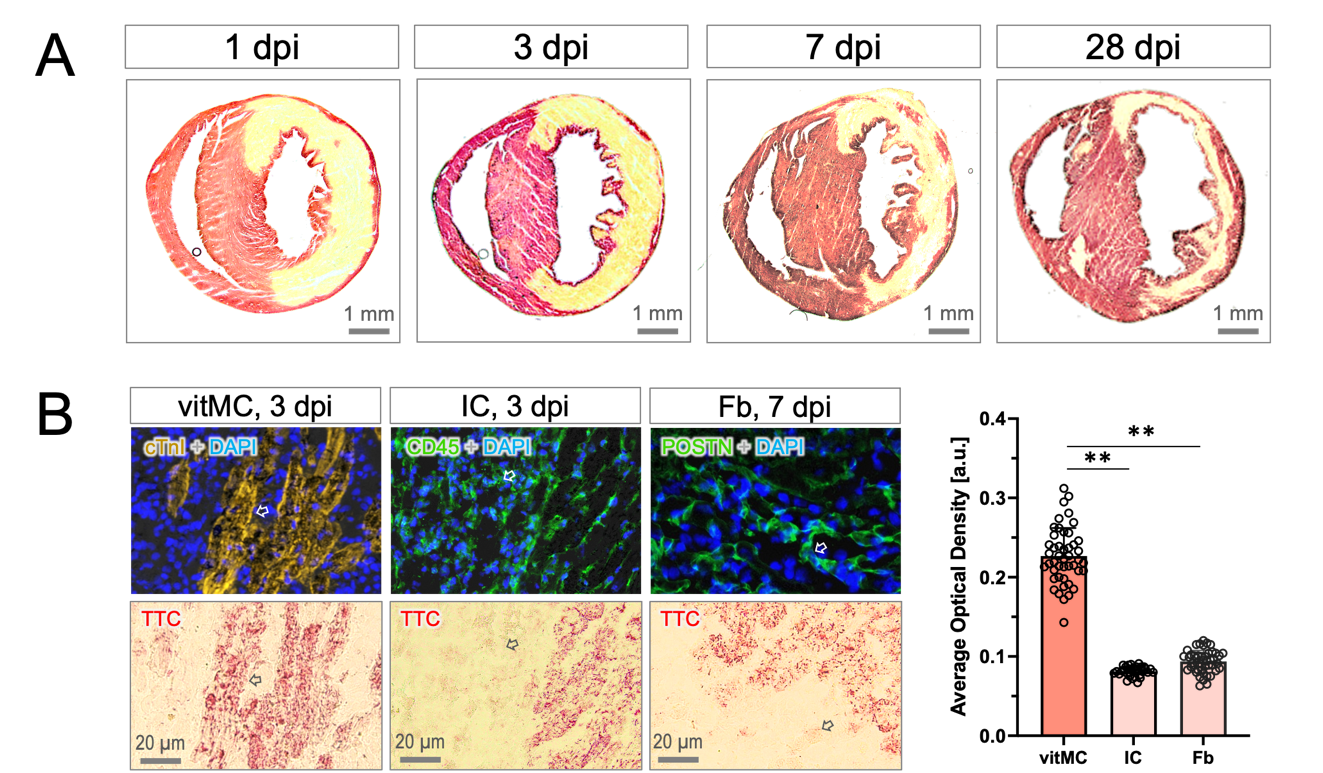


**Suppl. Fig. 2.** Application of the redTTC assay over an extended time frame. **A.** Heart samples were harvested at various time points, covering the acute phase at 24 hours and an extended time frame up to 28 days post infarction (dpi), and were stained using an identical protocol. The refined TTC assay consistently discriminates the infarct area where vital myocardium (vitMC) is lost at all time points. A significant thinning of the infarcted wall is observed in the chronic/old MI heart. **B.** Immunostaining in heart sections confirms the TTC signal preferentially identifies viable cardiomyocytes (cTnI positive, n=46) by the significantly dense formation of formazan precipitates, compared to vital immune cells (IC, CD45 positive, n=31) and fibroblasts (Fb, POSTN positive, n=45), which show relatively weak and faint optical density. Arrows illustrate the examples of the locations of positive cells in the immunostaining and the corresponding TTC images. ** indicates p < 0.01.


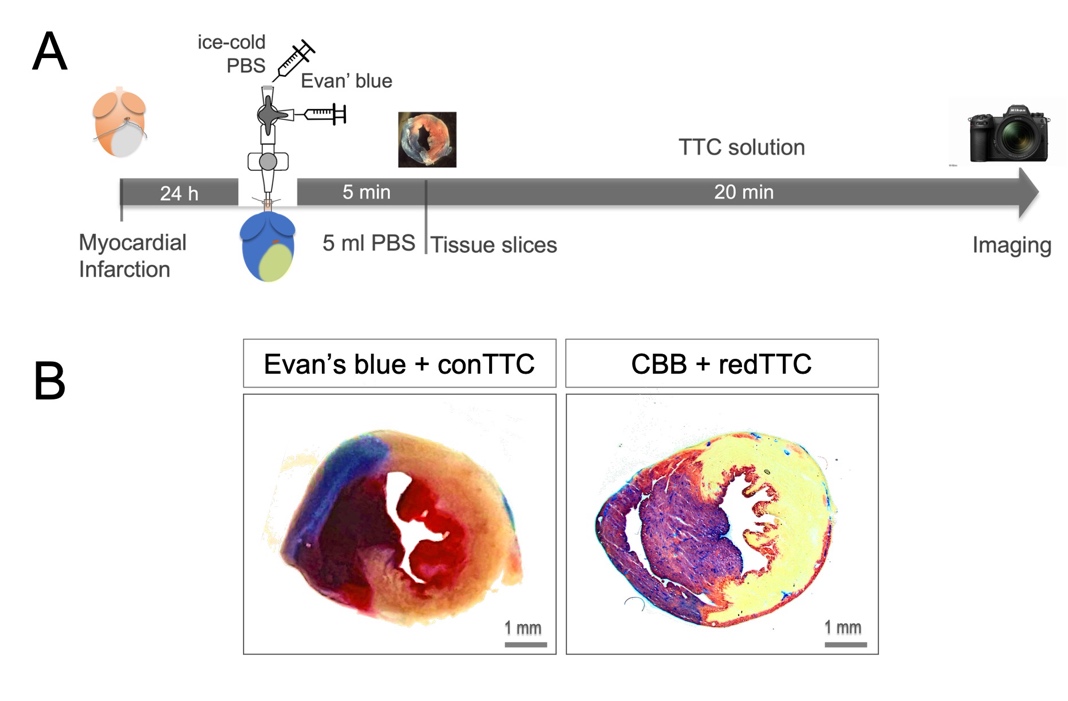


**Suppl. Fig. 3.** Detection of area-at-risk in 24-hour MI hearts by two methods. **A.** In the conventional method, Evan’s blue was given via the aortic cannula, and after a brief wash with PBS, tissue sections were made at 1-mm thickness. TTC staining was then performed by immersing the tissue slices in 1 % TTC solution at 37°C for 20 minutes. **B.** Gross histology was performed on tissue stained with the conventional TTC staining (conTTC, left). In the refined method (redTTC), Coomassie brilliant blue R250 (CBB) was combined with redTTC staining, and the heart sample was processed into a thin slice (50 µm, right). The protein-binding properties of CBB yield a clear-cut, unambiguous border that precisely defines the area-at-risk.

**
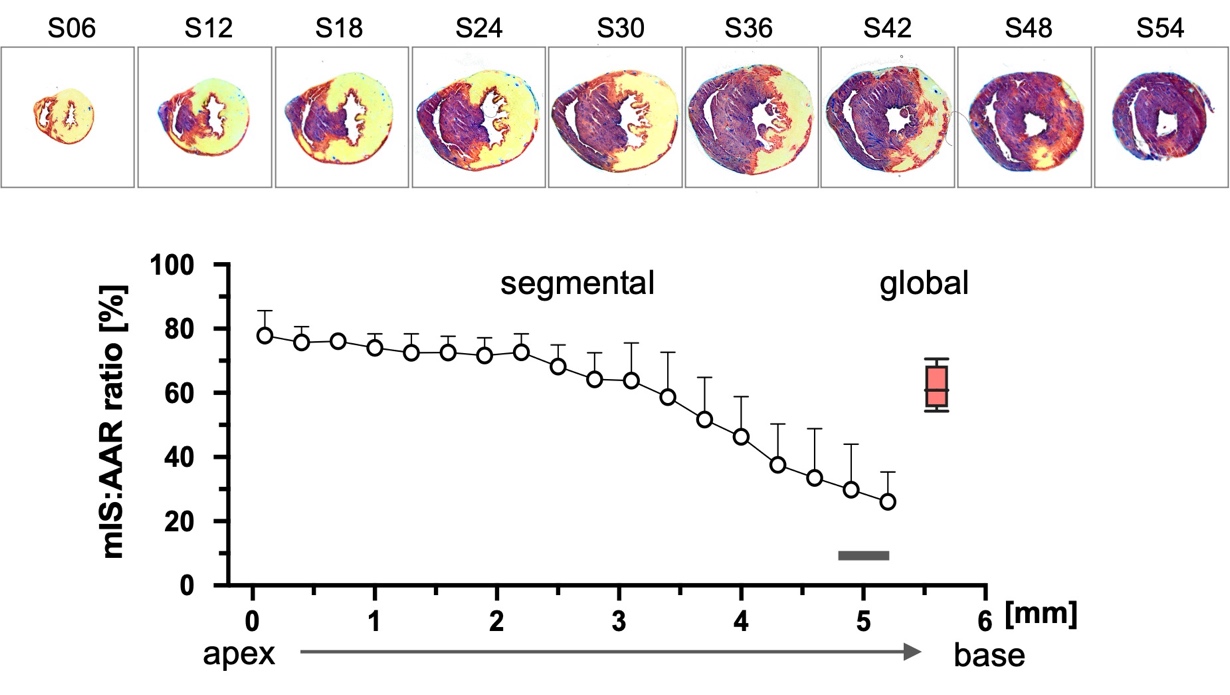
**

**Suppl. Fig. 4.** The segmental and global ratio of mIS:AAR. The segmental ratio was derived from the successive heart slices after CBB + redTTC duo-staining and presented at intervals of 300 µm, starting from the apex to the site of occlusion (indicated by bar, n=5). The ratio is relatively constant up to the mid-ventricular level and gradually declines until the occlusion site, primarily due to the collateral survival of cardiomyocytes. The box and whiskers plot indicates the global ratio of mIS:AAR calculated by volumetry.
